# A Horn-fed Frequency Scanning Holographic Antenna Based on Generalized Law of Reflection

**DOI:** 10.1038/srep31338

**Published:** 2016-08-12

**Authors:** Dawei Liu, Bo Cheng, Xiaotian Pan, Lifang Qiao

**Affiliations:** 1 School of Electronic and Information Engineering, Beihang University, Beijing, 100191, China

## Abstract

A new method of designing horn-fed frequency scanning holographic antenna is proposed. The artificial surface design of holographic antenna is based on generalized law of reflection. The input admittance is utilized to construct the interference pattern of the surface which is intervened by the excitation wave and the required radiation wave. The scalar admittance unit cell which is composed of sub-wavelength metallic patch on grounded dielectric substrate is implemented to design artificial surface, and the simulation results are just as expected that the antenna can scan the beam as the frequency changes. Furthermore, a cross shaped patch printed on grounded dielectric unit cells is used to reduce the designing complexity of tensor admittance surface. At last, a frequency scanning holographic antenna with tensor admittance surface with ability of changing linear polarization excitation wave to left-hand circular polarization (LCP) radiation wave is designed and fabricated. The full-wave simulation and experimental results show well agreement and confirm the method proposed.

Holography is an imaging technology when it was firstly proposed in 1948^1^. In the past few years, due to the appearance of metasurface and the development of surface plasmon polaritons (SPPs), holographic technique can achieve many novel functions, such as designing polarization and wavelength sensitive holographic surface by utilizing the polarization response of nano-structured elements^2^,^3^,^4^, detecting the orbital angular momentum of light^5^, modulating amplitude and phase of visible light by ultrathin hologram^6^, and creating holograms based on the optical scattering of plasmonic nanoparticles^7^. In 2011, the generalized laws of reflection and refraction were introduced to control electromagnetic wave flexibly^8^, and from then on, a so-called gradient index metasurface was presented based on the similar idea, and it can be used to convert propagating waves to surface waves^9^, achieving high-efficiency anomalous reflection^10^, and realizing ultrathin lenses with high efficiencies in transmission mode in the visible spectrum^11^.

With the rapid development of optical holography, the possibility of realizing microwave holographic antenna was verified in 1968^12^, and it was designed later^13^,^14^,^15^,^16^. Recently, the holographic leaky wave antenna with artificial impedance surface developed from SMRS (sinusoidally-modulated reactance surface) which was proposed in 1959 as a way of fabricating high gain surface wave antenna^17^ was investigated^18^,^19^,^20^. This type of antenna used the principle of interferometry in the designing process and defined the surface impedance of unit cell with sub-wavelength as the interference pattern, and then, the holographic artificial impedance surface was developed. The scalar and tensor impedance can be retrieved from the artificially structured surface^20^,^21^,^22^. In addition to the high gain characteristics, the designed surface was endowed with other functions, such as polarization controlling antenna using the tensor impedance surface^20^, surface wave propagation controlled by tensor impedance surface^23^,^24^,^25^, and 1-D and 2-D frequency beam scanning^21^,^26^. However, the microwave holographic antenna with feed-horn wave that has the function of frequency scanning has been studied few. At present, the grating reflector antenna has been utilized to achieve beam steering of linear polarization wave^27^ and circular polarization wave^28^, but the frequency scanning range is small.

In this article, a new way of designing holographic antenna with feed-horn wave having broad frequency scanning range is proposed. In the beginning, the basic theory of the generalized law of reflection and holographic antenna is introduced. And then, after some analyses about the characteristics of input impedance and the effect of the modulation depth and average impedance value on the antenna, we define the input admittance as the interference pattern. Then, the holographic antenna with the scalar admittance surface is constructed and simulated. Furthermore, we apply the tensor admittance unit cell to the holographic antenna in order to change the linear polarization incident wave from the feed to circular polarization radiated wave, and obtain a broad frequency scanning from the artificial admittance surface. For each tensor unit cell, there are three variables to be determined, and which are correlated to each other, so it is complicated to construct this artificial admittance surface. In this study, we put forward a method and choose an appropriate unit cell to reduce the designing complexity. At last, the holographic antenna with tensor admittance surface is fabricated and measured, it has a good performance on scanning beam in a broad frequency range, and it can also radiate circular polarization wave.

## Results

### Basic theory

**Figure 1:**
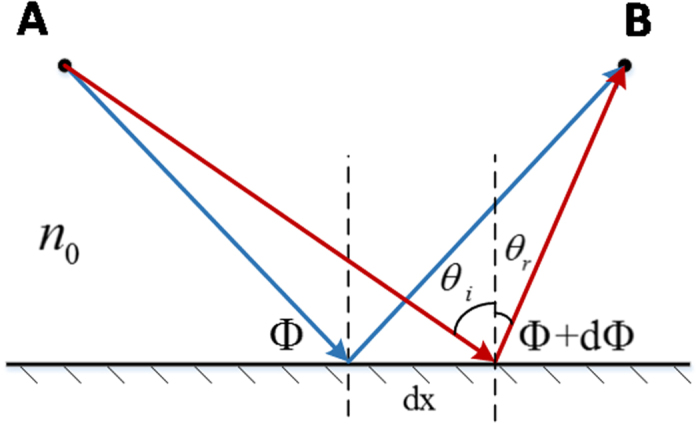
The schematic of the generalized law of reflection.

As the generalized law of reflection introduces^8^, when the incident wave with angle *θ*_*i*_ reaches the plane of reflection, and suffers a gradient phase discontinuity, an anomalous reflection will occur as shown in Fig. 1. And the equation of generalized law of reflection can be written as


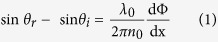


where *θ*_*r*_ is the angle of reflection, Φ and Φ + dΦ are the phase discontinuities at two points and *dx* is the corresponding distance. *n*_0_ is the refractive index of the upper half space and *λ*_0_ is the vacuum wavelength.

**Figure 2:**
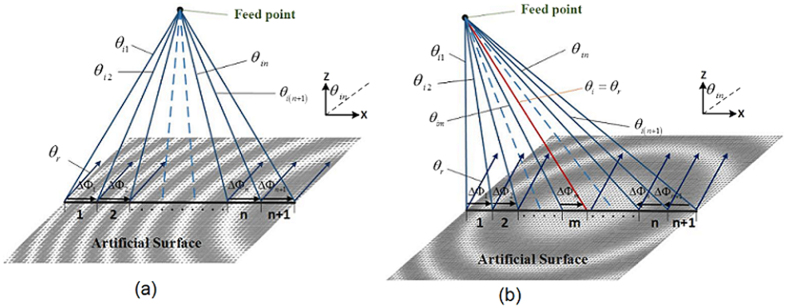
The schematic diagram of artificial surface in X-Z plane. (**a**) The reflected beam angle *θ*_*r*_ is larger than maximum of *θ*_*in*_. (**b**) The reflected beam angle *θ*_*r*_ is less than maximum of *θ*_*in*_ and larger than the minimum of *θ*_*in*_.

The generalized law of reflection can be applied to design the artificial surface to control the radiated wave of horn-fed holographic antenna and obtain a broad frequency scanning range. The feed-horn wave of the holographic antenna can be considered as radiation from a point in its far field region, and for different locations on the artificial surface, the incident angle of the reference wave varies. Here, we consider the two-dimensional case and divide the surface into many small units with same distance *d* as shown in Fig. 2. The beam generated by the feed illuminates the artificial surface, and then is reradiated by the surface. For a certain beam radiating direction *θ*_*r*_, the reflection of each artificial surface unit should be the same as following.


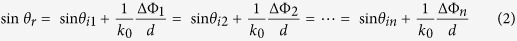


where *θ*_*in*_ is the incident angle of the reference wave and ΔΦ_*n*_ represents the discontinuous phase of the *n*th unit. *k*_0_ is wavenumber of free space. It is clear that *θ*_*in*_ and ΔΦ_*n*_ vary with the locations of the surface. Since the lattice spacing is much smaller than the wavelength, the phase gradient 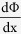 can be replaced by the difference 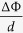.

When the reflected beam angle *θ*_*r*_ is larger than maximum incidence angle *θ*_*in*_, as shown in Fig. 2(a), for every unit cell in artificial surface, the sine function of the radiation beam angle can be written as


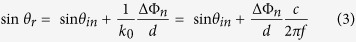


If the working frequency turns to *f*_1_ = *f* + Δ*f*, and the phase difference changes slightly, (*f* + Δ*f*)^−1^ can be expressed by Taylor series, and Eq. (3) will change to


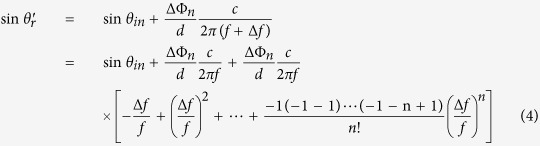


It can be seen that if the change of phase difference ΔΦ_*n*_ with frequency can be ignored, and Δ*f* is less than *f*, i.e., the rest items 
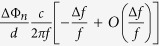
 will not change much, the reflection angle 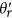 for every unit cell can be considered to be equal in spite of the frequency change, which also satisfies the condition as Eq. (2). So the beam direction of radiated wave will scan as frequency changes, and not diverge. When the frequency increases, the radiation angle will decrease.

If the reflected beam angle *θ*_*r*_ is less than maximum incidence angle *θ*_*in*_ and larger than the minimum incidence angle *θ*_*in*_, as shown in Fig. 2(b), the surface can be divided into two parts. One is the area with incident wave angle *θ*_*im*_ < *θ*_*r*_ and the other is the area with incident wave angle *θ*_*in*_ > *θ*_*r*_. The radiation direction in the two parts can be written as


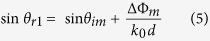



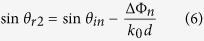


At the center frequency, due to the modulation of ΔΦ_*n*_, sin *θ*_*r*1_ is equal to sin *θ*_*r*2_. If the frequency deviates from the center frequency, the radiation angle of these two parts will have an inverse change, which causes the fact that the antenna can not scan its beam. It should be noted that if the working frequency is within the antenna bandwidth, the difference between *θ*_*r*1_ and *θ*_*r*2_ is small. So these two beams will not be separated, and they have the same radiation direction with the beam in the center frequency, but which causes the fact that the gain will decrease and the sidelobe level will get higher.

In this study, the SMRS is used to generate the surface phase gradient. As to the artificial impedance surface leaky wave antenna, the excited surface wave is commonly used as the reference wave. And the surface impedance which is defined as the ratio of tangential electric field to tangential magnetic field of the surface wave is modulated to construct the interference pattern, which is the interference of excited wave Ψ_*ref*_ and radiated wave Ψ_*rad*_. Where Ψ_*ref*_ is the reference wave, and Ψ_*obj*_ is the object wave that we are interested in. Because the holographic antenna is excited by the space wave in this study, the surface impedance can not be used to describe the surface’s electromagnetic characteristic any longer. We notice that the input impedance is the ratio of tangential electric field to tangential magnetic field of the space wave near the surface, so we define the input impedance as the interference pattern.


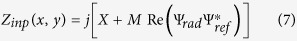


For a holographic antenna with a feed source of reference wave has height *H* away from the impedance surface, the reference wave can be written as


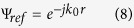


and the radiation wave which is in the X-Z plane and has an emitted angle *θ* down from Z axel can be written as


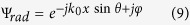


where 
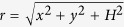
 is the radial distance of the feed and *φ* is an arbitrary initial phase.

Different from surface impedance, the input impedance always has a resonant point with the size changing of the metal patch at a certain frequency, and at this point, the impedance will be infinite and has a sharp rise or fall when the size changes few. If we set the average impedance *X* at the resonant point, the modulation depth *M* chosen can be quite large and therefore increase the radiation efficiency. For this reason, it is not appropriate to select an infinite constant to be the value of *X*. However, it can be noted that the infinite constant of input impedance corresponds to a zero value of input admittance, so the input admittance can be used in the design of holographic antenna with feed-horn wave. It shows that if the admittance and impedance are modulated in the same form, they will have the similar structure nature. As a result, we use input admittance to design holographic antenna and define it to be the interference pattern as


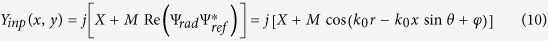


where *X* is the average value of input admittance and equals to zero. *M* is the modulation depth, and 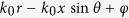 corresponds to the phase discontinuity.

### Holographic antenna with scalar admittance surface

In this chapter, two holographic antennas with different interference patterns are designed to illustrate the frequency scanning based on the generalized law of reflection. The designed beam angle of antenna 1 is fall in a range between the maximum and minimum incident angle of the excited wave, and the antenna can not scan its beam. And antenna 2 is that the emitted beam angle is larger than the maximum incident angle, and the antenna can scan the beam as frequency changes. The two artificial admittance surfaces are excited by TM polarization wave and the radiation wave from two surfaces has the same polarization. Antenna 1 has an offset feed horn pointing the center of the surface by an incident angle 30° with a height of 24 cm, and the angle of outgoing beam is also 30°. The feed horn of antenna 2 has a height of 20 cm and illuminates the center of the surface perpendicularly. The angle of outgoing beam is 45°. Both of the two antennas work in the center frequency of 15 GHz.

The unit cell of the scalar admittance surface that we choose is the square metal patch printed on the grounded substrate and has a period of 4 mm. The material and thickness of the substrate between the two surfaces are different. The first surface has a dimension of 28 × 28 *cm*^2^, containing 4900 unit cells. And the second surface has a dimension of 20 × 20 *cm*^2^, containing 2500 unit cells.

**Figure 3:**
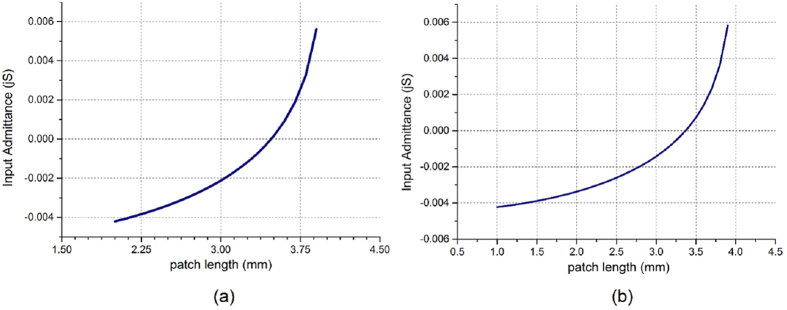
The input admittance calculation results. (**a**) The unit cell of antenna 1. The permittivity of the substrate is 2.2, and the thickness is 1.57 mm. The incident angle is 30°. (**b**) The unit cell of antenna 2. The permittivity of the substrate is 2.5, and the thickness is 1.57 mm. The incident angle is 0°.

**Figure 4:**
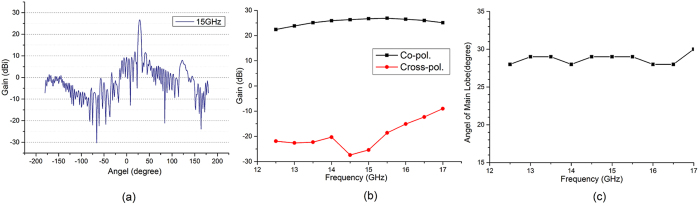
Simulation results of antenna 1. (**a**) The radiation pattern in the center frequency. (**b**) The co- and cross-polarization gain in multiple frequencies. (**c**) The angle of main lobe in multiple frequencies.

**Figure 5:**
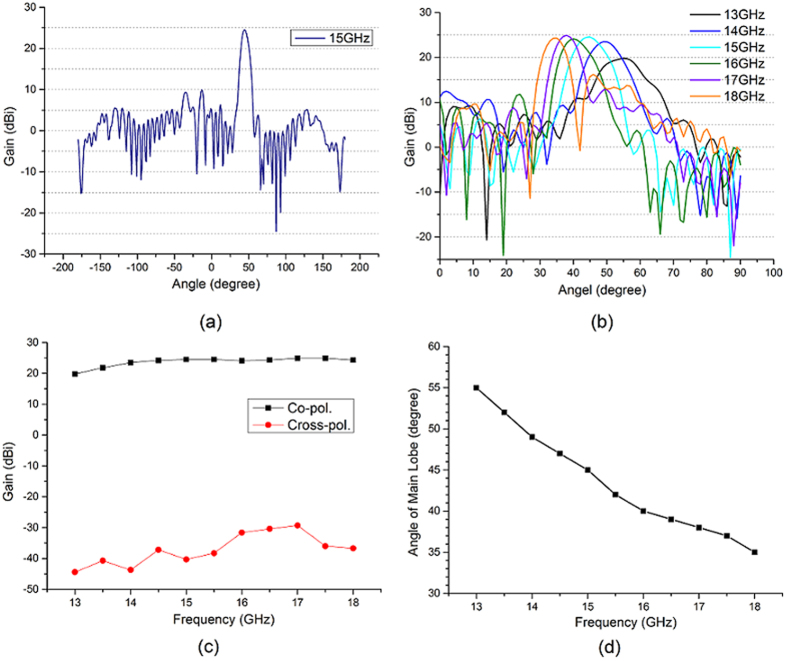
Simulation results of antenna 2. (**a**) The radiation pattern in center frequency. (**b**) The radiation patterns in the multiple frequencies. (**c**) The co- and cross-polarization gain in multiple frequencies. (**d**) The angle of main lobe in multiple frequencies.

We have calculated the input admittance of the two unit cells with different patch size as shown in Fig. 3. Both of the two antennas’ modulation depth is 0.004. The full-wave simulation results of these two holographic antennas in multiple frequencies are shown in Figs 4 and 5, respectively. From Fig. 4 we can see that antenna 1 has a good main lobe in the frequency range of 12.5–17 GHz, and the cross polarization level can reach −52 dB at the center frequency 15 GHz. This antenna has a stable beam direction within the bandwidth. As predicted, antenna 2 can scan its beam with frequency changing, the angle can be swept from 55° to 35° between 13 GHz and 18 GHz. We can also calculate the radiation direction in each frequency by Eq. (4). Assuming sin *θ*_*in*_ = 0, the calculated beam direction from frequency 13 GHz to 18 GHz is 55°, 49°, 45°, 42°, 39° and 36°. They have well agreement with the simulation results and the little difference may be caused by the deviation of the phase distribution at different frequencies. And the antenna also has a good cross polarization level of −64.8 dB at the center frequency.

### Holographic antenna with tensor admittance surface

In addition, the tensor admittance surface is implemented to the holographic antenna to achieve the function of changing the linear polarization excited wave to circular radiated wave with the frequency scanning characteristic.

Based on Ampere circuit law, the tensor input admittance relates the tangential electric to the surface current density as


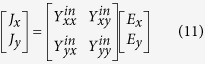


A LCP radiated wave with an incidence angle *θ* in the X-Z plane will be studied, so the form of surface current density 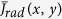 can be written as


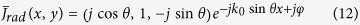


where *φ* is an arbitrary initial phase. The electric field of the excited reference wave can be written as


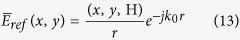


Then, we can get the interference pattern of the tensor input admittance, similar with tensor impedance expression Eqs (19) and (20) in ref. 20.


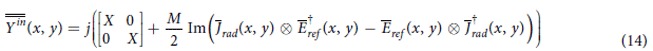


where † means Hermitian conjugate, *X* is the average admittance and equals to zero, and *M* is the modulation depth. There are three variables 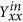, 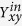 and 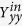 to be determined in one unit cell. Furthermore, there are thousands of cells in one admittance surface, and every unit has special patch structure, so it is complicated to determine the three variables. We need to use a new procedure and structure to design artificial surface. We have known that if the coordinate axis is rotated by angle *φ* along the clockwise direction, the input admittance will turn into^29^


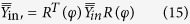


The expression of *R*(*φ*) is


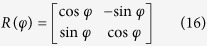


We can diagonalize each unit’s input admittance to obtain the two admittances in the principle axis directions and the rotation angle *φ*_*rot*_. Then, the variables are reduced to the two admittances in the principle diagonal of matrix.

**Figure 6:**
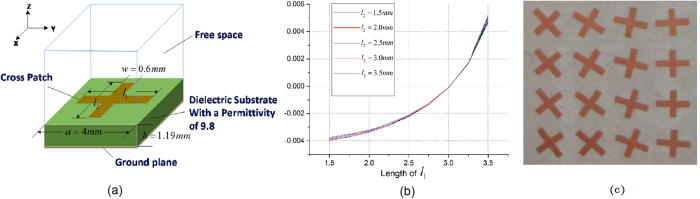
(**a**) Tensor admittance unit cell. The period of the dielectric substrate is *a* = 4 *mm* and thickness is *h* = 1.19 *mm*. The width of the cross arm is *w* = 0.6 *mm*, and we can control the arm length *l*_1_ and *l*_2_ to change the input admittance in the orthogonal direction. The substrate material we choose is Taconic CER-10 with a permittivity of 9.8. (**b**) The input admittance in Y axis of the unit cell. The operating frequency is 13.5 GHz, and the length of *l*_2_ varies between 1.5 mm to 3.5 mm. (**c**) The fabricated unit cells.

The unit cell that we use here is the cross patch printed on the grounded dielectric, as shown in Fig. 6(a). It can be seen that the patch has an orthogonal shape and the extraction result of input admittance in principle Y axes direction is shown in Fig. 6(b). From this figure, we can see that for a certain length *l*_1_, the changes of input admittance with length *l*_2_ are negligible, and we can consider that the admittances in the orthogonal directions are uncorrelated. Thus, for each unit cell, the lengths of *l*_1_ and *l*_2_ corresponding the diagonal matrix of admittance can be extracted from Fig. 6(b), and the cross patch can be rotated by angle −*φ*_*rot*_, then the cell can be determined. The above method will simplify the designing procedure of admittance surface structure significantly.

### Experimental results

**Figure 7:**
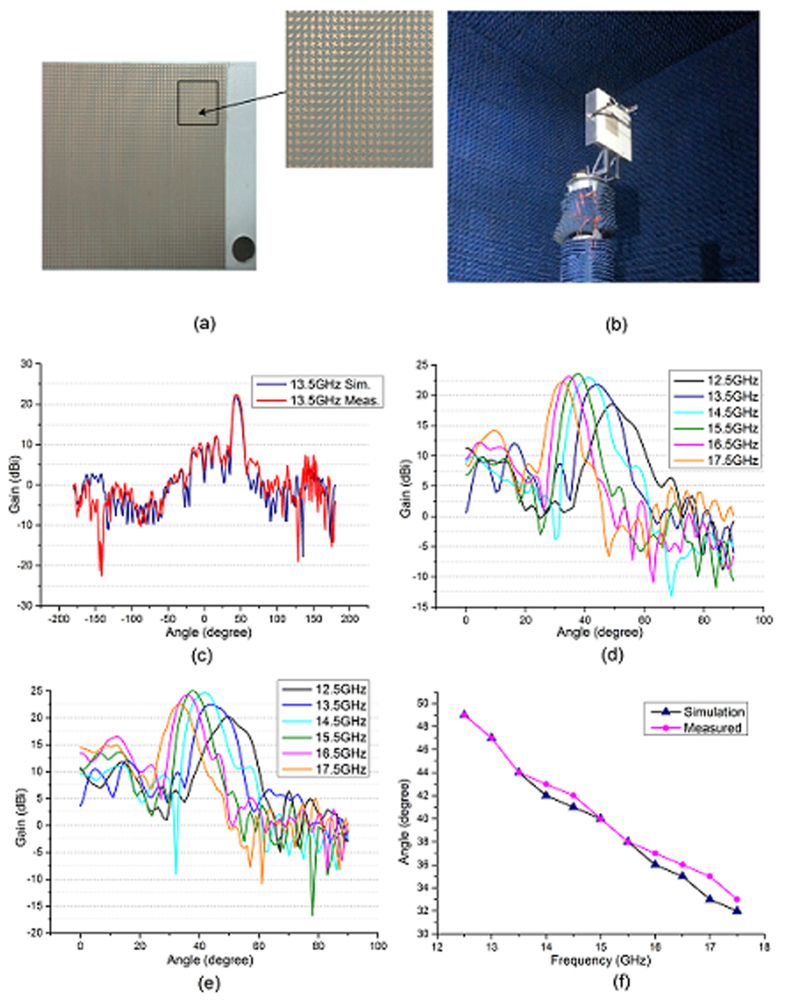
(**a**) The fabricated admittance surface. (**b**) The experimental setup of the antenna. (**c**) The simulation and measurement LCP radiation pattern in the center frequency. (**d**) The simulation LCP radiation pattern in multiple frequencies. (**e**) The measurement LCP radiation pattern in multiple frequencies. (**f**) The main lobe angle in multiple frequencies.

The circularly polarized antenna that we designed contains 50 × 50 unit cells, and the feed is pointing the center of artificial surface by an incident angle 0° with a height of 20 cm. The modulation depth is 0.0032, and the angle of outgoing beam is 45° at the center frequency 13.5 GHz. The configuration of the antenna is shown in Fig. 7(a,b). This antenna was assembled and measured in an anechoic chamber. During the measurement, the antenna was mounted on a turntable and it was away from the reflector of the compact field by a distance about 7 m. The two feed horns of the antenna and compact field were connected to the S1 and S2 port of vector network analyzer respectively. The simulation and measurement results are presented in Fig. 7(c–f). From Fig. 7(c–f), we can see that the measurements has well agreement with the simulations. We also calculate the beam directions at frequency ranging from 12.5 GHz to 17.5 GHz by Eq. (4). They are 50°, 45°, 41°, 38°, 35° and 33°, respectively. The theoretical analysis has almost no difference with the simulation and measurement results. However we still find some deviation between simulations and measurements. For instance, the sweep range is 16°, a slightly smaller than the simulation result 17°. The reason is that the artificial surface is pasted on a board and there are some necessary metal brackets to support the board and the feed horn, which will have effects on the measurement results since they are not contained in the simulation model. In addition, the feed’s assembly error and artificial surface’s machining error will also influence the measurements.

## Discussion

We presented a new method of designing horn-fed frequency scanning holographic antenna by implementing admittance surface based on the generalized law of reflection. The scalar and tensor input admittances were used to construct the interference pattern. Three antennas were designed in this article. The first one is not a frequency scanning antenna, which has a stable beam direction in a frequency range of 12.5 GHz–17 GHz. The second one can scan beam from 55° to 35° as frequency changes from 13 GHz to18 GHz. The last one with tensor admittance surface structure was fabricated and measured, and it shows well agreement with simulation results, which can scan the beam by 16° as frequency changes and change the linear polarization excited wave to circular polarization radiated wave. These antennas have larger scanning angle range than the frequency scanning grating reflector antenna proposed before.

## Additional Information

**How to cite this article**: Liu, D. *et al*. A Horn-fed Frequency Scanning Holographic Antenna Based on Generalized Law of Reflection. *Sci. Rep.* **6**, 31338; doi: 10.1038/srep31338 (2016).
